# CD8^+^ Cytotoxic-T-Lymphocyte Breadth Could Facilitate Early Immune Detection of Immunodeficiency Virus-Derived Epitopes with Limited Expression Levels

**DOI:** 10.1128/mSphere.00381-18

**Published:** 2019-01-09

**Authors:** Tetsuo Tsukamoto, Hiroyuki Yamamoto, Tetsuro Matano

**Affiliations:** a Department of Immunology, Kindai University Faculty of Medicine, Osaka, Japan; b AIDS Research Center, National Institute of Infectious Diseases, Tokyo, Japan; c The Institute of Medical Science, The University of Tokyo, Tokyo, Japan; Beth Israel Deaconess Medical Center

**Keywords:** AIDS, cytotoxic T lymphocyte, human immunodeficiency virus, parallel processing, simian immunodeficiency virus

## Abstract

Cytotoxic-T-lymphocyte (CTL) responses are important to control the replication of human immunodeficiency virus (HIV) and simian immunodeficiency virus (SIV). Accumulating evidence suggests that the ability of a few immunodominant T-cell populations to detect and kill HIV/SIV-infected cells is important in individuals with a protective major histocompatibility complex class I (MHC-I) allele. On the other hand, immunization with live(-attenuated) viruses may be effective against superinfection of virulent viral strains regardless of the host’s MHC-I haplotypes, although the underlying mechanisms have not been fully documented. In this article, we propose a hypothesis that the early detection of infected cells in superinfected individuals may be partly facilitated by recognition of diverse CTL epitopes with limited expression levels. We further explain the hypothesis using simple mathematics that was written based on previous *in vitro* viral suppression assay results and by considering the physical contact of infected cells with CTLs.

## Abstract (precis)

Cytotoxic-T-lymphocyte (CTL) responses are important to control the replication of human immunodeficiency virus (HIV) and simian immunodeficiency virus (SIV). Accumulating evidence suggests that the ability of a few immunodominant T-cell populations to detect and kill HIV/SIV-infected cells is important in individuals with a protective major histocompatibility complex class I (MHC-I) allele.

## OPINION/HYPOTHESIS

### Immunodominance and breadth in the CTL-based control of immunodeficiency virus.

CD8^+^ cytotoxic-T-lymphocyte (CTL) responses are important to control the replication of human immunodeficiency virus (HIV) and simian immunodeficiency virus (SIV). However, the mechanisms underlying the cellular immune response for the optimal efficacy of CTLs remain unclear. Specific major histocompatibility complex class I (MHC-I) alleles, such as the human leukocyte antigen HLA-B57, enable the excellent control of HIV-associated viremia based on immunodominant CTL responses that target only a few epitopes, which results in the selection of an escape mutation that can be a significant fitness cost to the virus (1). CTL-based prophylactic vaccines can be effective for subjects who carry these epitopes, referred to as elite controllers (2), while an alternative strategy, such as the persistent induction of effector memory CTL responses, is necessary for those who do not carry the HIV-resistant HLA-I allele (3). In such a case, the antigen expression levels on host cells should be strictly controlled for the maintenance of functional T-cell populations, because antigen abundance may easily cause T-cell exhaustion (4).

In contrast to an immunodominant CTL response, a broad CTL response targeting diverse epitopes derived from a wide range of HIV antigens may also be effective, although the mode of action may be different from that of a narrow immunodominant CTL response (5). For example, a mosaic HIV-1 vaccine optimized for the broad coverage of T-cell epitopes has been tested in a phase 1/2a clinical trial (APPROACH) and highly successful (6, 7). The high efficacy of a broad CTL response in the suppression of HIV/SIV replication has been explained as the ability to suppress the replication of CTL escape variant viruses (8). Although this holds true in the presence of CTL escape mutations, the idea does not explain a potential benefit of broad CTL responses that are induced early in infection and support viral control in the absence of a CTL escape. This article was aimed to propose an alternative way to stoichiometrically explain the ability of a “broad CTL response” to suppress viral replication.

### *In vitro* viral suppression efficacy of CD8^+^ cells obtained from live-immunized macaques.

In a previous study, Burmese rhesus monkeys were vaccinated with a vector or vectors transiently expressing SIV antigen. They were then challenged with the highly pathogenic CXCR4-tropic simian/human immunodeficiency virus (SHIV) strain SHIV89.6PD, which expresses the SIVmac239 Gag and Pol and HIV-1 89.6PD Env proteins. In contrast to unvaccinated naive monkeys that developed massive viremia and acute symptoms of AIDS following the infection, viral replication was controlled in vaccinated monkeys (9). Vaccination also stringently controlled the superchallenge with CCR5-tropic SIVmac239 with no or little viremia in peripheral blood, in contrast to the usual failure to control primary SIVmac239 infection in unvaccinated naive rhesus monkeys (10).

**FIG 1:**
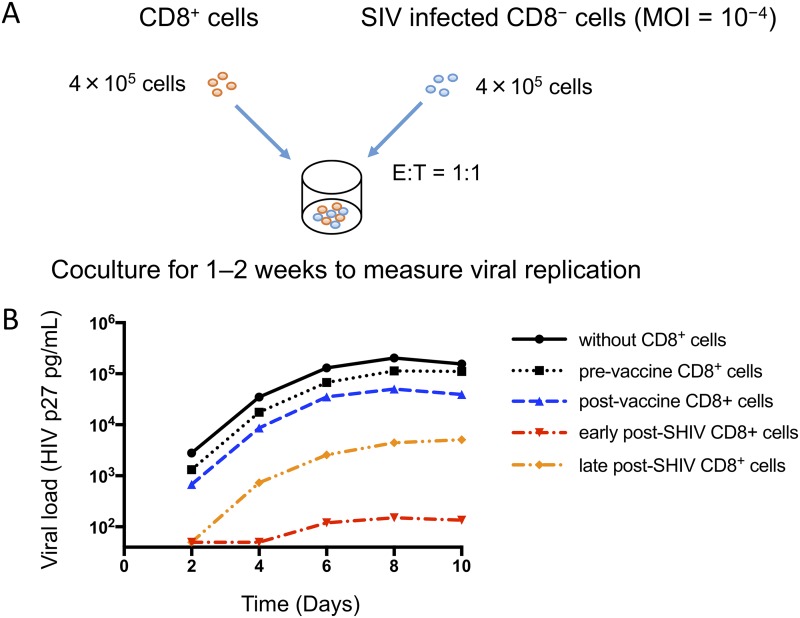
A summary of previous *in vitro* VSA results using rhesus macaque PBMCs. (A) A schematic representation of the *in vitro* VSA method. (B) Representative results of the VSA using bulk CD8^+^ cells. PBMCs were collected before and after transient vaccination as well as at the early and late stages of infection after live immunization with SHIV89.6PD. Bulk CD8^+^ cells were isolated using CD8 magnetic beads and tested for their efficacy to suppress SIVmac239 replication *in vitro*. SHIV89.6PD and SIVmac239 both code for Gag and Pol but not Env. The efficacy of CD8^+^ cells obtained after live immunization was better than that of naive or transiently vaccinated CD8^+^ cells with viral load differences of approximately 10^2^- to 10^3^-fold. The data were adapted from our previous publication with kind permission (10).

Because the SHIV genome persisted in superchallenged monkeys, the superchallenge virus, a virulent SHIV strain, was assumed to serve as an immunogen against SIVmac239. In a *post hoc* analysis, peripheral blood mononuclear cells (PBMCs) of superchallenged monkeys were collected before and after vaccination with the transient vector and at the early and late stages of infection after the SHIV challenge. As described in Fig. 1, the ability of bulk CD8^+^ cells isolated from PBMCs was tested for its efficacy to suppress the replication of SIV *in vitro*. The *in vitro* viral suppression assay (VSA) clearly demonstrated that the ability of live-immunized CD8^+^ cells to suppress SIV is much better than that of unvaccinated or vaccinated CD8^+^ cells (10). However, the underlying mechanisms are still unclear.

### A description of an *in vitro* VSA using simple mathematics.

**FIG 2:**
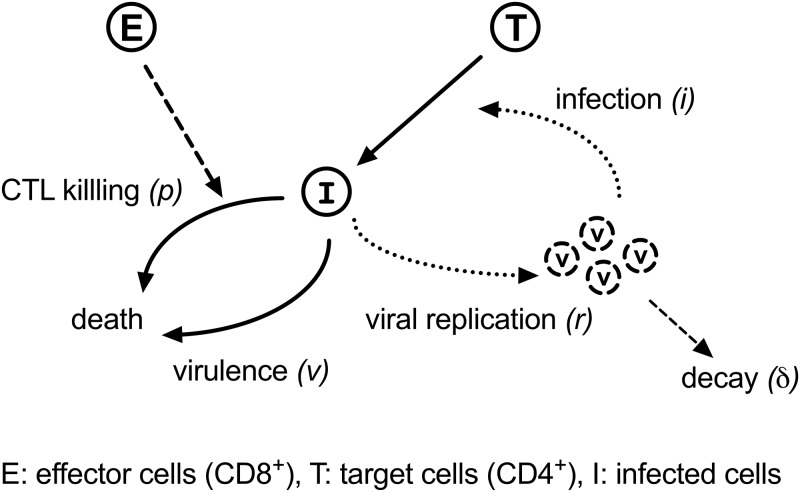
A schematic representation of viral replication and suppression *in vitro*. Target cells (*T*) are infected with virus (*V*) at the rate *i*. Infected cells produce the virus at the rate *r* and are killed by the virus at the rate *v* or by CTLs at the rate *p*. Virus decay occurs at the rate δ. The proliferation rates of cells are omitted from the scheme and its mathematical description because cells are cultured in the presence of IL-2 and quickly grow to confluence following phytohemagglutinin stimulation of target cells for 48 h (10).

Well-established ordinary differential equation (ODE) models for simulating viral replication in mammalian cells exist (11). In Fig. 2, target cells (*T*) are infected with the virus (*V*) at the rate *i* and become infected cells (*I*), which produce the virus at rate *r*, but infected cells are killed by the cytopathic effect of the virus at the rate *v*. Virus decay occurs at rate δ. A mathematical representation of Fig. 2 ignoring cell proliferation is as follows:

(1) $$\frac{dT}{dt}=iVT$$

(2) $$\frac{dI}{dt}=iVT-vI$$

(3) $$\frac{dV}{dt}=rI-\delta V$$

Then, the above mathematical expression was modified to include CTLs as an effector. CTLs detect and kill infected cells (*I*) at the rate *p*. The viral replication/suppression *in vitro* is illustrated in Fig. 2. Equation 2 was modified to include the CTL-killing rate *p* as follows:

(4) $$\frac{dI}{dt}=iVT-vI-pI$$

For simplicity, an effector-to-target (*E*/*T*) ratio of 1 is assumed in the following discussion (Fig. 1). It was also assumed that each cell is surrounded by 12 other cells in a close-packed structure. The average number of CTLs surrounding an infected cell can be regarded as follows:

(5) $$K=12\cdot \frac{E}{\left(E+T+I\right)}$$

We propose to predict the killing rate *p* based on the assumption that multiple CTLs work in parallel to detect and kill infected cells. If the number of CTLs recognizing the infected cell is *K*′(≤*K*), then, the killing rate *p* in equation 4 is expressed as follows:

(6) p=ρK′

given that ρ is the killing rate per CTL. In equation 6, *p* can be smaller than ρ*K* if the epitope expression level is not sufficient for *K* of CTLs, meaning that some of the surrounding CTLs (*K* − *K*’) fail to recognize the infected cell. We then considered epitope expression levels on an infected cell. Besides the parameters in the above equations and Fig. 2, *q* was defined as the viral production rate necessary for CTL stimulation. Given that *r* represents the viral production rate per infected cell, the number of CTLs recognizing an infected cell (*K*’) is *r*/*q* (≤*K*).

**FIG 3:**
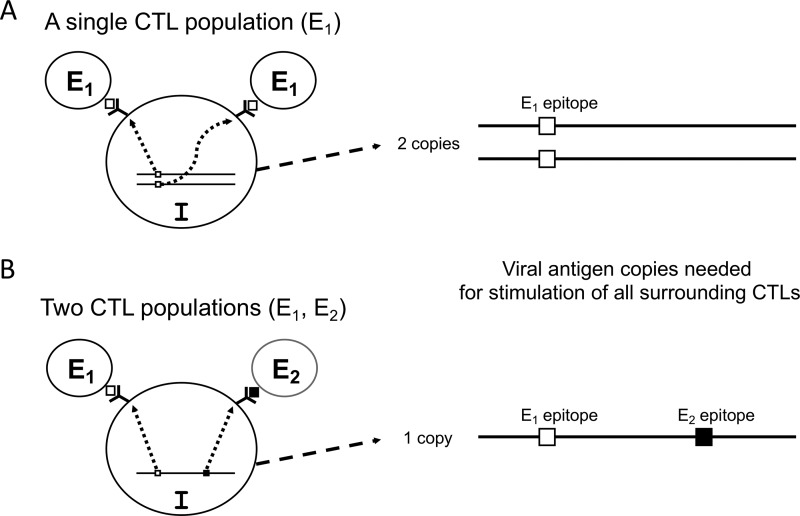
The concept underlying the proposed parallel processing hypothesis. Epitopes are presented by MHC molecules and supposed to be mobile according to the fluid mosaic model. (A) Given that an infected cell (I) is surrounded by two CTLs (*E*_1_) that recognize the same epitope, infected cells produce viral antigens to express the amount of epitope necessary for recognition by both CTLs. (B) If the surrounding two CTLs (*E*_1_ and *E*_2_) recognize different epitopes derived from different regions of viral antigens, the required rate of viral antigen production for CTL recognition will be lower; thus, the CTL response will be initiated earlier.

Next, we considered multiple CTLs recognizing different epitopes on an infected cell. According to Fig. 3, the rate of viral production necessary to simultaneously stimulate two or more CTLs differs depending on the number of epitopes recognized by the CTL population. Our hypothesis supposes that there are *n* different epitope-specific CTL populations and that the epitopes are encoded in different parts of the viral genome. In the following discussion, almost all differences between the CTL populations (e.g., killing rates and cell counts) are ignored except epitope specificity.

If the CTL response is sufficiently broad (*n* ≥ *K*), then the lowest rate of viral production necessary to stimulate *K* CTLs, each recognizing different epitopes, can be only *q*. On the other hand, to stimulate *K* CTLs recognizing the same epitope (*n* = 1), the infected cell needs to allow for a greater viral production rate by *K*-fold (*Kq*), resulting in a higher viral load as the CTL response is not optimized for the detection of infected cells in which the virus replicates slowly. Taken together, the number of CTLs that an infected cell can stimulate (*K*’) is *nr*/*q* (≤*K*) or *K*. Therefore, to represent the killing rate (*p*), equation 6 can be rewritten as follows:

(7) $$p=\rho \cdot \min\left\{n\frac{r}{q},K\right\}$$

**FIG 4:**
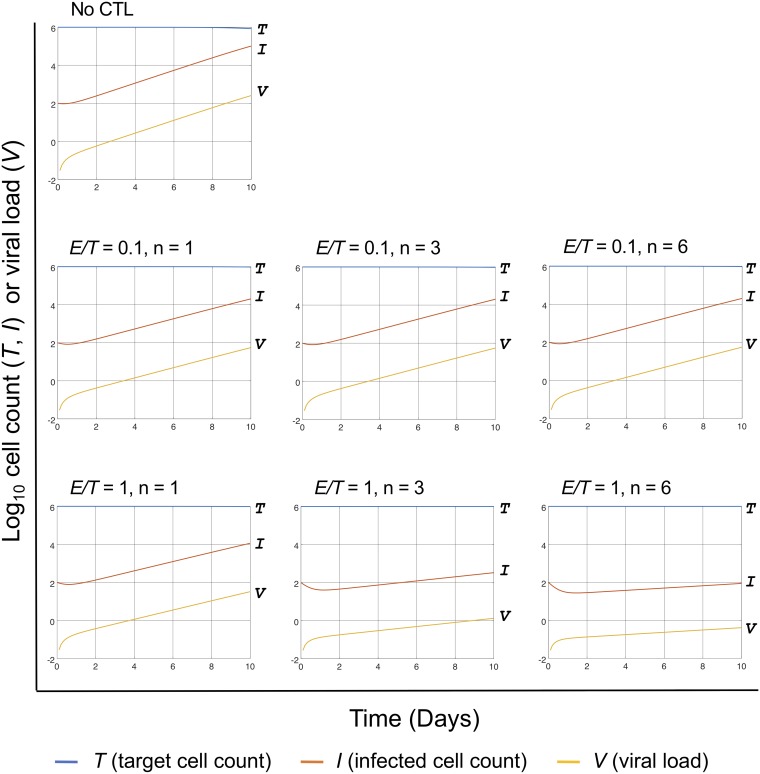
Sample output calculated from the mathematical expression of the hypothesis. Values along the *y* axes are presented in a log scale. At time zero, the values of *T* (total cell count), *I* (infected cell count), and *V* (viral load) were set to 999,900, 100, and 0, respectively, meaning that the multiplicity of infection was set to 10^−4^. The initial *E/T* ratio was set to 0.1 or 1. Parameters *v* (=0.4 cells/day) and δ (=0.5 viral particles/day) were set according to reference 12, while *r* (=0.003 ng/day) was set according to reference 13 and based on the assumption that 10^4^ SIV particles contain about 1 pg of p27, as is the case with HIV-1 p24. Parameter *i* (=0.0005 ml/ng day) was set by roughly fitting to previous *in vitro* viral replication data (10, 14). Values for ρ and *q* were set to 0.3 and 0.002, respectively, as an example.

**FIG 5:**
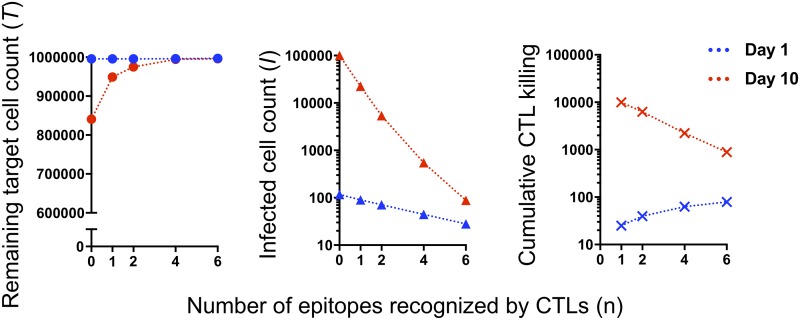
CTL breadth may affect both the efficacy and efficiency of killing. Remaining target cell counts (*T*, plotted in a linear scale, left), infected cell counts (*I*, plotted in a log scale, middle), and cumulative killing by CTLs (plotted in a log scale, right) at days 1 (blue lines) and 10 (red lines) after infection are calculated and compared between different numbers of CTL epitopes recognized (*n*). *n* = 0 means results with no CTL.

**FIG 6:**
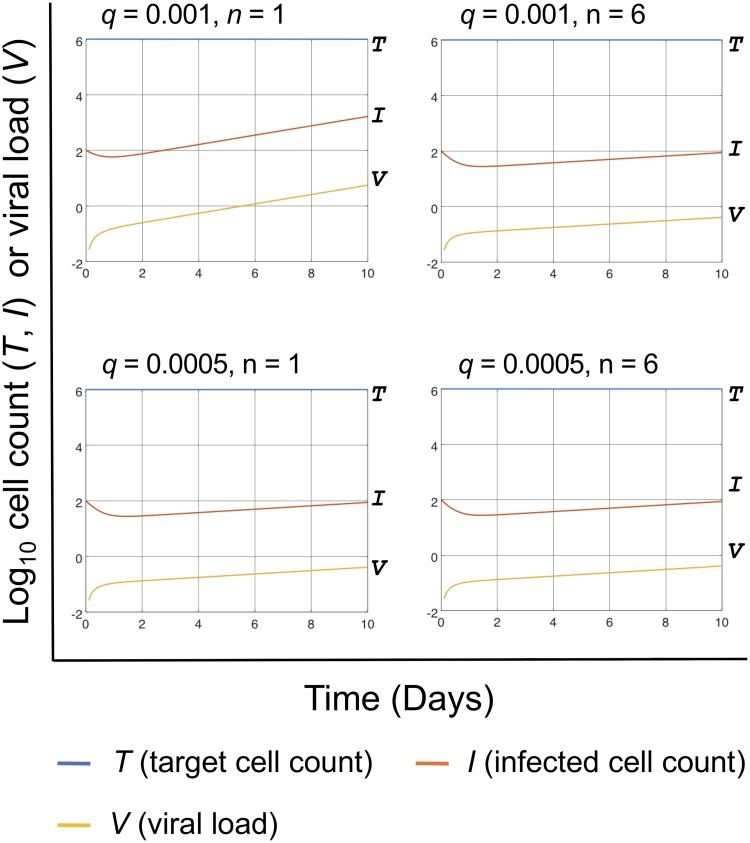
Higher sensitivity of CTL may reduce the effect of CTL breadth on killing. Output was calculated from the same equations as in Fig. 4, with almost the same parameter settings except that the initial *E/T* ratio was fixed to 1, while *q* (antigen amount necessary for recognition by one CTL) was set to either 0.001 or 0.0005 (compared to 0.002 in Fig. 4) and *n* (CTL breadth) was set to either 1 or 6. Note that lower *q* means higher sensitivity of CTLs to detect their specific epitopes.

Sample results were calculated from equations 1, 3, 4, 5, and 7 as shown in Fig. 4. The values of some parameters were set based on those in previous reports (12, 13). The output for *n* = 0, i.e., in the absence of CTLs, roughly matched that of previous studies (10, 14). In addition, the remaining target cell counts (*T*), infected cell counts (*I*), and cumulative counts of infected cells killed by CTLs at day 1 and 10 after infection are shown in Fig. 5. In Fig. 6, we further demonstrate the effect of CTL breadth on viral suppression when CTLs have higher sensitivity (i.e., lower *q*) than in Fig. 4.

In our hypothesis, CTLs with different epitope specificities cooperated to detect viral infection early and, therefore, can better limit viral replication without increasing the *E/T* ratio (Fig. 4). Furthermore, recognition of more epitopes caused higher killing rates at day 1 postinfection but resulted in lower cumulative killing by CTLs at day 10 (Fig. 5), implicating higher efficiencies and less likelihood of T-cell exhaustion in the long run. The proposed mechanism may explain the viral control achieved in the superinfection of SIV following the live(-attenuated) SIV/SHIV immunization of rhesus macaques, where virus-specific T cells are supposed to be trained and optimized for the vaccine strain that persists and slowly produces viral proteins (10, 15, 16).

### Possible ways to demonstrate parallel processing of multiple epitope information.

CTLs have been typically viewed as efficient killer cells (17). However, recent studies have reported that the efficacy of individual CTLs might be somewhat limited (18). Moreover, the cooperation of multiple CTLs to kill an infected cell has been reported (19). It has been shown that weak T-cell responses against many graft leukocyte-derived peptides collectively facilitate allograft rejection (20). These findings further confirm the basis of our hypothesis (Fig. 6) and suggest possible involvement of CTL breadth in determining the threshold viral replication levels in latently infected HIV reservoirs. Considering the physical properties of lymphocytes that are more or less round, the current hypothesis is based on our extensive discussion on previous *in vitro* assay results and assumption that, if cells are placed in a close-packed structure at the *E/T* ratio of approximately (≈) 1, then each infected cell will be surrounded by ≈12 different cells (≈6 target cells and ≈6 CTLs; the real *E/T* ratio is applied to the calculations as shown in equation 5), and assessed by the surrounding CTLs recognizing different (up to ≈6) epitopes. The close-packed structure adopted here may somewhat represent the cellular arrangements not only *in vitro* but in lymphoid organs. However, because of the mobility of CTLs necessary for systemic immune surveillance *in vivo*, the breadth of CTLs may have an even higher impact *in vivo* on the early detection and killing of infected cells (21). This notion may be further emphasized by the fact that HIV-infected target cells themselves show high cellular motility across differential lymph nodes (22).

The relationship between epitope expression levels on antigen-presenting cells and T-cell interclonal competition in proliferative responses has been previously described (23). The authors hypothesized that high epitope expression levels result in a response by immunodominant T cells, whereas low epitope expression levels allow for the coexistence of different T-cell populations that broadly cover the antigen. The present hypothesis is logically associated with this previous study. Taken together, these two theories indicate that antigen-limited settings help induce broad CTL responses, and that those broad CTL responses are really optimized for detecting infected cells with limited epitope expression levels (Fig. 3). The exhaustion of T cells in viral antigen abundance may also be considered in the future (Fig. 5) (4).

More information on the viral replication rate (*r*) is essential to better understand its relationship with epitope expression levels. Although the viral replication rate in equation 3 was set at a constant value for simplicity, it can be a function having multiple variables such as integrated viral copy number (a factor uniquely important for retroviruses), epigenetic modification, and cell activation status. Accordingly, it is predicted that cells with low integrated viral copy numbers, or cells with low viral replication rates (e.g., latently infected cells) will require a broad CTL response for surveillance. In this regard, the current hypothesis described in Fig. 3 can serve as a platform for further sophistication in future studies. One major interface to incorporate in the model may be initial kinetics of dendritic cell (DC)-mediated CTL antigen priming.

In general, chronically infecting viruses, such as HIV, can be compared with acutely infecting viruses, such as influenza viruses. However, the former can intrinsically avoid T-cell recognition by their low replication rates; therefore, a broader CTL response is needed for better control. On the other hand, the latter replicates more rapidly, but it is more prone to immune recognition and, therefore, is more dependent on the magnitude, rather than the breadth, of the virus-specific CTL response. However, further studies are needed to confirm these results. Coexistence of other immune effectors, such as virus-specific neutralizing antibodies, may potentially modulate the repertoire/breadth of CTL responses in both settings (24).

The demonstration of the proposed parallel processing hypothesis in wet *in vitro* assays is possible by using several populations of HIV or SIV epitope-specific CTLs as follows.

- Prepare ≈6 epitope-specific CTL populations that recognize different parts of viral antigens but are similar in their killing profiles when tested individually. Optionally, the virus (HIV or SIV) may be genetically modified to express all of these epitopes at similar levels.

- Prepare human or simian CD4^+^ T cells (possibly a cell line) expressing all the MHC-I molecules to interact with all of the above epitope-specific CTLs.
- Perform viral suppression assays similar to the one described in Fig. 1 with a fixed total CTL count (such as 4 × 10^5^ cells) but with different numbers (*n* = 1, 2, …, 6) of epitope-specific CTL populations put in the cocultures with CD4^+^ T cells as shown in Fig. 4.

However, particular efforts and care will be necessary to make the assays sufficiently practical and scientific, because of different properties with different epitopes/CTLs (e.g., epitope expression levels, avidities, CTL proliferation rates, and so forth) that may make data highly ambiguous in the current technical setting. We are also aware that the experimental observations in superchallenged macaques could be affected by other factors, including MHC-I haplotypes and the induction of effector memory CTLs by prime-boost effects. Future studies will thus need to exclude these factors. Because of the current difficulty in developing a method that satisfies all these conditions, we believe it is reasonable to present the hypothesis and expect this article to stimulate discussion on the subject. We expect that not only virology and immunology but biophysics and informatics may be helpful for future analyses.

In summary, a hypothesis was proposed that incorporates an adaptive immune response that takes the best advantage of the information coded in the viral genome by checking multiple CTL epitopes, particularly in the early stage of SIV superinfection following immunization with live(-attenuated) strains when epitope expression levels are still limited. Our description of the hypothesis is based on insights obtained from previous *in vitro* assay results and may further apply to more complex *in vivo* events. Furthermore, the high tendency of persistence-prone and latently infecting viruses being equipped with virulence proteins specialized for downregulating MHC-I antigen presentation (25, 26) may be empirical evidence of them building a major bottleneck against this host surveillance system. In addition to the recursion-based virus-specific naive CD4^+^ T-cell depletion hypothesis that we recently presented (27), the parallel information processing mechanism proposed here promotes a shift in our thinking regarding the design of adaptive immunity in terms of both efficacy and efficiency.
